# Unravelling the Impact of Diverse Fermentation Techniques on Key Nutrient Absorption in Bambara Groundnut and African Yam Bean: A Review

**DOI:** 10.3390/foods15061109

**Published:** 2026-03-23

**Authors:** James Elegbeleye, Dharini Sivakumar

**Affiliations:** 1 Department of Crop Sciences, Tshwane University of Technology, Pretoria 0001, South Africa; elegbeleyeja@tut.ac.za; 2 Centre for Nutrition & Food Sciences, Queensland Alliance for Agriculture and Food Innovation, The University of Queensland, Brisbane, QLD 4108, Australia

**Keywords:** African yam bean, Bambara groundnut, legumes, food security, fermentation, bioavailability, antinutritive factors (ANFs), neglected and underutilised species (NUS)

## Abstract

Amid growing concerns about climate change and its potential impacts on food security and malnutrition, there is a need for climate-smart crops to help mitigate these challenges. African yam bean (*Sphenostylis stenocarpa*) and Bambara groundnut (*Vigna subterranea*) are considered climate-smart neglected or underutilised species (NUS) in sub-Saharan Africa (SSA). These legumes are rich in nutrients, comprising fats, carbohydrates, and protein, as well as essential micronutrients. However, their use is constrained by the presence of antinutritive factors (ANFs) such as oxalates, tannins, and phytates, which reduces mineral bioaccessibility and protein digestibility. Fermentation provides a cost-effective means of effectively reducing these antinutrients, thereby making these crops more mainstream due to their enhanced bioavailability and bioactivity. This review summarises the impact of diverse microbes and fermentation techniques on the bioavailability of essential nutrients in Bambara groundnut and African yam bean. The importance of pre-treatment steps such as soaking, germination, dehulling, and thermal treatment will also be discussed. By synthesising recent studies, the review explores the mechanisms by which fermentation degrades the ANFs, enhances nutrient bioavailability and improves protein digestibility from these crops. This review explores the pivotal roles of fermenting microbes, such as species of *Lactobacillus* and *Bacillus*, during the process of biotransformation.

## 1. Introduction

Despite the significant progress made globally in combating micronutrient deficiency, Africa has experienced no remarkable changes, with an increasing challenge of malnutrition, marked by significant variations across regions and countries. Different demographics, especially children, pregnant women, and the elderly, are often the most affected [1]. Bambara groundnut (*Vigna subterranea*) (BGN), originating from the Bambara tribe of Mali, and African yam bean (*Sphenostylis stenocarpa*) (AYB) are climate-smart, nutrient powerhouse, underutilised legumes with significant potential to mitigate the malnutrition problem as well as other dietary deficiencies, especially in Africa and other regions facing food insecurity. Bambara groundnut and African yam bean are native to sub-Saharan Africa (SSA), with observed richness in complex carbohydrates, proteins, fibre, unsaturated fatty acids, and essential minerals (magnesium, iron, zinc, and potassium) [2,3]. Both crops are resilient to harsh climates and require little agricultural input, which further makes them suitable for cultivation in resource-limited settings. The nutritional profiles of these legumes, combined with their adaptability, position them as strategic crops in the fight against dietary deficiencies [4].

However, in SSA, where a significant part of the population is affected by malnutrition, the presence of antinutrients in these legumes significantly reduces the nutrient bioavailability, further limiting their adoption in mitigating these crises [5]. Despite their nutritional richness and potential, the uses of these crops are limited by the presence of antinutritional factors (ANFs) such as phytic acid, tannins, trypsin inhibitors (TIs), and polyphenols. These compounds can bind essential minerals, such as iron, calcium, and zinc, to form insoluble complexes, thereby reducing their bioavailability and impairing nutrient absorption [6]. In some cases, antinutrients interfere with protein digestibility, further limiting the nutritional value of these legumes. The effects of ANFs on human nutrition are significant because they not only diminish the uptake of minerals, such as iron (Fe) and calcium (Ca), but can also contribute to metabolic dysregulation via chronic mineral deficiency. An example of this is a situation where the ANF lectin is present at elevated levels, leading to a disruption of the intestinal integrity, potentially resulting in an autoimmune response [7]. For instance, phytic acid chelates divalent cations, whereas tannins form complexes with proteins, carbohydrates, minerals, and digestive enzymes. The ANF TI directly hinders proteolytic activity in the gastrointestinal tract. Consequently, ANFs reduce both macro- and micronutrient availability, potentially exacerbating metabolic dysregulation [6]. This biochemical hurdle to nutrient bioabsorption underlines the importance of different processing techniques that are efficacious in reducing concentrations of ANFs without compromising the sensory or physicochemical properties of the final product [2].

Fermentation is one of the traditional, most effective low-cost processing practices for enhancing the bioavailability of nutrients in legumes. By nutrient bioavailability, we mean the in vivo absorption and utilisation of these nutrients, while bioaccessibility and *in vitro* protein digestibility (IVPD) are merely laboratory proxies. Fermentation leverages the metabolic capabilities of microbes, resulting in the biotransformation of compounds and the production of organic acids that lower the pH levels in the substrate. Lactic acid fermentation involves acidification (pH < 5) and endogenous phytase activation, while alkaline *Bacillus* fermentation relies on proteolysis and ammonia production at higher pH (8.5–9). The low-pH milieu of lactic acid fermentation promotes the solubilisation of minerals and also reduces the chelation properties of some ANFs [8]. Furthermore, microbial fermentation activates endogenous phytase via acidification and enzyme induction. The activated phytase breaks down phytic acid to produce inositol phosphates with comparatively lower affinity for minerals [7]. In addition, in the course of the fermentation process, macromolecules are broken down into simpler, more accessible forms, including protease inhibitors, thus enhancing digestibility [9]. Other key benefits of fermenting these legumes generally also include the improvement of sensory attributes and consumer acceptability of legume-based products. A good example of this is when a conventional cereal ingredient is substituted with Bambara groundnut in fermented beverages. This was demonstrated to result in a lower concentration of phytate while enhancing protein quality and flavour profiles, such as in fermented BGN yoghurt-like beverages [10,11]. Such composites are typically relevant in the development of complementary foods intended for infants and the young, who require a nutrient-dense, highly digestible diet for critical growth and development [2].

Beyond microbial fermentation, complementary pre-treatment and selective breeding have been explored for their ability to reduce ANFs [12]. Soaking, as a pre-treatment step, aids the leaching of water-soluble ANFs, such as some phenolics, as well as the activation of the endogenous enzyme phytase [13]. Roasting, on the other hand, denatures heat-sensitive protease inhibitors due to moisture loss, but on a dry-weight basis, it primarily affects quality and digestibility [14]. Similar to roasting, treatment with cold plasma (for 10–20 min at low power) significantly reduces the concentration of phytates, without causing extensive damage to nutrients, as observed with thermal processing [15]. There are genetic approaches, including breeding for low-ANF varieties, that select for favourable traits while maintaining yield stability [12]. Studies have reported wide ranges of tannin concentrations across genotypes, with some showing strong positive correlations between condensed tannins and trypsin inhibitor activity [16].

The broader context of food security in sub-Saharan Africa (SSA) underscores the urgency of this review, as numerous households lack consistent access to balanced diets containing sufficient levels of protein, minerals, vitamins, and other essential nutrients. For instance, the prevalence of malnutrition is alarmingly high (the highest globally) in the Democratic Republic of Congo (DRC), with at least 50% of the population deficient in essential nutrients [17]. Underutilised legumes like AYB and BGN present a sustainable solution due to their adaptability to marginal soils and low-input farming systems. However, their full potential can only be realised if the challenges posed by antinutrients are addressed through effective bioprocessing, such as fermentation. By reducing ANFs while enhancing the bioavailability of nutrients using post-harvest processing interventions, these underutilised legumes are positioned to play a greater role in mitigating malnutrition across vulnerable populations [2,3].

Despite the increasing research, there are crucial gaps in understanding how fermentation enhances the nutrient bioavailability of these legumes. Unlike AYB, there are robust studies on the nutritional profiles of BGN and the effects of lactic and alkaline fermentation [10,18], whereas the studies on AYB are usually limited to compositional analyses with fewer trials on fermentation conditions (e.g., spontaneous vs. controlled) and variability in the protein digestibility [3,19]. The existing gap lies in direct comparisons of lactic acid vs. alkaline fermentation of these legumes, specifically under standardised conditions. This will furnish essential information such as the ANF:mineral ratio (for predicting the chelation inhibition), simulated mineral uptake (using Caco-2 cells), and in vivo validation of bioavailability using human or animal models using serum micronutrient levels after consumption. Therefore, this review addresses the gaps by synthesising the previous studies into a framework for these legumes while, at the same time, recommending standardised metrics for future research in a way that bridges mechanistic and applied knowledge. We will also consider the scalability of the products within local contexts where indigenous knowledge intersects with modern food science innovations. This integrated approach will facilitate the translation of these products into valuable components in functional foods, complementary feeding programmes, and sustainable agricultural systems for communities that rely on these legumes as staple foods.

## 2. Methodology

We consulted academic and multidisciplinary databases including Google Scholar, Scopus, Web of Science (core collection), PubMed/MEDLINE, CAB Abstracts, AGRICOLA and Food Science and Technology Abstracts (FSTA) as well as specialised repositories (such as CGIAR CGSpace, IITA Genetic Resources Center, FAO) for this review based on their comprehensive coverage of microbiology, nutrition, agriculture and food science topics using Boolean operators (AND, OR, NOT). The literature search was conducted from July 2 to 28, 2025, using database-specific search strings. The process of screening took place in two phases. We had an initial pool of 450 records that were retrieved across the databases (PubMed n = 120, Scopus n = 150, Web of Science n = 100, Google Scholar n = 50, CAB Abstracts n = 30, AGRICOLA n = 10, FSTA n = 10, CGIAR/IITA/FAO (handpicked n = 10). After duplicate removal (n = 150), 300 unique records remained. Title and abstract screening were independently performed by two reviewers, and consultation was done with a third party where necessary. Ultimately, 85 studies met all criteria and were included in the narrative synthesis. The database-specific strings are as follows: For PubMed, the search combined terms related to fermentation techniques (fermentation techniques OR microbial fermentation OR lactic acid fermentation OR alkaline fermentation) with nutrient-related outcomes (nutrient absorption OR bioavailability OR antinutritional factors) and the target crops (Bambara groundnut OR *Vigna subterranea* OR African yam bean OR *Sphenostylis stenocarpa*). In Scopus, the equivalent TITLE-ABS-KEY structure was applied to the publication. For Web of Science, we employed the TS = topic search field. The Google Scholar search utilised an allintitle: query with date limitation set via advanced search settings, while for CAB Abstracts we combined the core concepts. Supplementary keywords that were used included *Bacillus*, *Lactobacillus*, neglected underutilised species, antinutrients, antinutritive factors, and mineral bioaccessibility. We emphasised studies that provide mechanistic insights and practical applications on African yam bean and Bambara groundnut, including book chapters, reviews, meta-analyses, and empirical studies from 2015 to 2025, including some highly cited foundational studies (with exceptions for key pre-2015 works). The selected studies are the ones directly addressing the impact of fermentation—alone or in combination with pre-treatments—on antinutritional factors, nutrient composition, protein digestibility, mineral bioaccessibility, or related outcomes in Bambara groundnut and/or African yam bean. The exclusion criteria eliminate studies that are not in English (English-only inclusion may under-represent local African literature), are not focused on the subject matter, lack verifiable data, are duplicates, or lack full-text access or rigorous methodology. Since this review is a narrative review and not a formal systematic review or meta-analysis, we did not apply any quantitative risk-of-bias scoring tool. However, qualitative consideration was given to study design rigour, sample size, replication, analytical methods, and reporting transparency when interpreting and weighting findings. We opted for a narrative review rather than a full systematic review because of the limitations in the evidence, heterogeneity and the exploratory nature of studies on the fermentation of these legumes. The narrative format provided a meaningful organisation of the fragmented literature, comparing what is available while highlighting the differential progress between the two legumes. This provided a clear, actionable framework for future research that is required in this field. It must be noted that the health-related claims in this review depend solely on in vitro data unless stated otherwise since no in vivo studies on fermented BGN or AYB were identified.

## 3. Nutritional Profiles and Antinutritional Factors of Bambara Groundnut and African Yam Bean

Both BGN and AYB possess a considerable amount of nutrients that position them as viable options for protein sources next to other well-known legumes such as soybean and cowpea. For instance, BGN is a nutrient-dense food with a balanced composition of macronutrients [20,21]. The observed ranges of some of the nutrients present in BGN and AYB, such as proteins and micronutrients, provide a significant contribution towards daily requirements. Specifically, BGN has about 15 to 25% (dry-weight basis) BGN protein content in the raw, mature seed. Some sources indicate a range from 9.60% to 40.0%. On average, BGN contains about 20.34% to 20.56% protein. This variation can be attributed to the genotypic differences, growing conditions, and the analytical techniques used for estimation [2,22]. The proteins in BGN overlap significantly with those of cowpea. But unlike cowpea, soyabean, and most legumes, the BGN amino acid score is unusually higher when compared with that of cowpea (80% BGN/74% soyabean/64% cowpea) [23]. The amino acid score is the ratio of the limiting essential amino acid in the protein (as a percentage of the requirement pattern) compared to a reference protein or FAO/WHO standard pattern. This gives BGN an edge and makes it an excellent complementary protein source when compared with cereals, which are often deficient in lysine but rich in methionine. The most abundant macronutrient is carbohydrates, with approximately 42.0% to 70.0% (dry-weight basis) of the seed, with starch accounting for a significant part (about 49.5%) of the total carbohydrates, depending on the genotype. Protein and carbohydrate are the major components of the seed. Fats constitute about 5–7%, predominantly consisting of unsaturated fatty acids, primarily oleic acid and linoleic acid (omega-6) [2,22,24]. The composition of essential mineral elements includes as potassium (K), ranging from 819.335 mg–1290 mg/100 g; magnesium (Mg) at 136.0 ± 2.0 mg/100 g; zinc (Zn) between 2 mg/100 g–5 mg/100 g; and lastly iron (Fe) ranging from 4 mg/100 g to 9 mg/100 g [3,21,25,26].

When compared with soyabean, BGN’s nutritional composition is based on its remarkable balance of macronutrients, along with the moderate fat content. Besides challenges such as the “hard-to-cook” nature of these legumes, they are excellent staple crops that can potentially deliver sustained energy and nutrition in a single source when consumed or used as functional ingredients [20]. On the other hand, the high protein and fat contents, along with a lower carbohydrate content, limit its function as a staple crop, but make it useful as a specialised or functional ingredient. BGN also contains numerous phytochemicals and bioactive compounds, contributing to its potential as a nutraceutical ingredient. Some of its phenolic compounds are predominantly located in the seed coats, with more relative abundance in seeds with dark or red colours. Some examples of the phenolics include flavonoids (e.g., quercetin, kaempferol, rutin, and myricetin), phenolic acids (e.g., caffeic acid, ρ-coumaric acid, and ferulic acid), and condensed tannins/proanthocyanidins (e.g., catechin and epicatechin), besides the bioactive peptides [6,27].

BGN and AYB both have strong potential for incorporation into complementary foods for routine infant and young child feeding (typically 6–24 months), where the objective is to provide balanced nutrition to support healthy growth, prevent micronutrient deficiencies, and complement breastfeeding or family foods [24]. In contrast to complementary food, ready-to-use therapeutic foods (RUTFs) are special lipid-based nutrient supplements that are used for outpatient children of 6 months or older with severe acute malnutrition (SAM) [28]. Although these BGN complementary foods align with the guidelines stipulated by FAO/WHO (400–425 kcal/100 g), they fall short of the RUTF standards (520–550 kcal/100 g). One study found that the carbohydrate content in BGN snack bars is sometimes above the RUTF guideline, with a lower fat content, which is regularly lower, indicating a need to increase fat content to meet RUTF fat standards [28]. Concerning the mineral contents, a 100 g dry serving of BGN typically supplies, using the WHO/FAO RNI, potassium (800–1400 mg), magnesium (120–200 mg) and zinc (2–5 mg), with reasonable amounts of iron (4–9 mg) and selenium. Additionally, BGN is very low in Na, positioning it suitable in line with public health advice, and its Fe content also aligns with the recommended daily allowances for adult males (10 mg) and pre-menopausal (19–50 years) women (15 mg) [29,30].

AYB is equally rich in protein, with observed amounts typically ranging from 19% to 30% and carbohydrates typically range between 49.88% to 63.51%, with a relatively low fat (principally α-linolenic and linoleic acid) content of between 1.39% to 7.53%, aiding weight management. The protein is reputed for its richness in essential amino acids, with the dominant fractions being albumin and globulin proteins [3]. AYB also contains important minerals such as K, Mg, Zn, and Fe in a proportion that is higher when compared to soya and common beans. The raw AYB is observed to contain 598.83 mg/100 g of K and 130.84 mg/100 g of Mg. Besides the essential micronutrients, AYB possesses bioactives that are important in mitigating certain lifestyle diseases, such as polyphenols and flavonoids [31,32]. Total phenolic content (TPC) is dependent on the processing the crop has undergone. For instance, TPC concentration ranges from 0.7 to 3.29 mg GAE/g in raw samples, while the germinated and fermented AYB samples tend to have higher TPC. The reason is that processing steps, such as fermentation, utilise the activity of microbial enzymes to hydrolyse and release bound polyphenolic compounds, thus enhancing the overall bioavailability [3,33].

Both BGN and AYB surpass RDIs for K, Mg, Zn, and Fe compared to soybean, which makes them superior in this regard. AYB is notably rich in Ca, Fe, and Zn [34]. The above nutritional profiles of BGN and AYB reveal compelling and valid reasons for the utilisation of these underutilised legumes as sustainable, nutrient-dense alternatives to the common legumes. However, on critical analysis, their efficacy in meeting Recommended Dietary Intakes (RDIs) is highly contingent on the processing steps they are subjected to, fortification, and targeted demographic application. Both BGN and AYB possess high concentrations of macronutrients, which make them suitable for incorporation into formulations such as complementary foods or ready-to-use therapeutic foods (RUTFs). Both legumes either meet or exceed international complementary feeding standards for key macronutrients such as protein and selected micronutrients per realistic serving, although they fall short of full RUTF and fat requirements. A notable example is the case of a BGN-derived snack bar, from a study by Adewumi et al. (2022) [28] with 14.1–14.8% protein and protein-calorie contributions (12.0–13.8% of energy), thus complying with the guidelines by WHO. The same trend was also observed in AYB, where lysine, tryptophan, and threonine in fermented AYB slightly surpassed the limits proposed by the WHO [3]. Generally, it has been observed that fermented AYB condiments are ideal for toddlers (2–5 years) because essential amino acids such as leucine, phenylalanine, tyrosine, valine, and isoleucine are higher in concentrations compared with WHO recommendations for children [24]. It must be noted, however, that the overall protein quality of BGN flour is below the recommended FAO/WHO value, necessitating dietary complementarity. This observed value means that BGN flour needs to be supplemented with other protein sources to meet ideal nutritional requirements, specifically in vulnerable populations [25,35].

BGN and AYB are highly nutritious legumes with proven potential to combat protein-energy malnutrition (PEM), especially in infant complementary foods, and micronutrient deficiencies, especially in sub-Saharan Africa (SSA), where this challenge is dominant. Their low fat gives them the edge in food applications that do not require defatting and contributes to a lower caloric value. The legumes’ favourable amino acid profiles and richness in minerals in processed form make them promising candidates in functional food and future food systems [3]. Nevertheless, the alignment with the stipulated RDI/RDA standards is conditional on the processing steps the legumes are subjected to. For these legumes to transition from promising alternatives to actual nutritional solutions, they must be methodically processed to reduce the concentrations of their antinutrients and strategically incorporated into fortified food blends tailored to specific demographic needs [36]. This is because the richness in the mineral content does not directly translate to high nutritional uptake or bioavailability. Hence, the actual nutritional value is not just a function of their composition, but of the science applied to make this bioavailable. A critical factor responsible for the reduction in bioavailability in these legumes is the presence of antinutritional factors (ANFs) or antinutrients [6]. Table 1 shows the nutritional composition and antinutrients present in BGN and AYB compared to the mainstream or common legumes.

### 3.1. Antinutritional Factors (ANFs)

Despite the potential of these legumes, antinutrients or antinutritional factors (ANFs) impair nutrient utilisation by complexing with divalent cations such as Fe^2+^, Zn^2+^ and Ca^2+^, reducing mineral bioaccessibility or directly inhibiting proteolytic enzymes, thus decreasing protein digestibility and amino acid release. When these ANFs are consumed at elevated concentrations, the outcome can negatively impact health. The main ANFs associated with BGN and AYB include phytic acid/phytates, tannins, trypsin inhibitors, and oxalates. The primary mechanism of action of these ANFs is the chelation of essential minerals and inhibition of digestive enzymes. For instance, phytate is linked to anaemia through Fe chelation [2,3].

Phytate, also known as myoinositol hexakisphosphate (IP6), is one of the main forms of storage of phosphorus (P) and inositol in these legumes, often contributing up to 75% of the total concentration of P. The strong binding or chelating nature of the molecule is ascribed to the polyanionic form conferred by the six phosphate groups [37]. Phytate exerts its action by strongly chelating multivalent cations, such as Zn, Ca, Mg and Fe, in the upper gastrointestinal tract, forming insoluble phytate–mineral complexes. These phytate–mineral complexes can neither be digested nor absorbed by the body. Humans and other monogastric animals produce limited intestinal phytase; the breakdown of phytate during fermentation occurs primarily through endogenous phytase present in the seeds and microbial phytase produced by the fermenting microbes as well as minor contributions from colonic microbiota. This outcome reduces the absorption and, in turn, the bioavailability of the essential micronutrients from the chyme. Besides the chelating nature of phytate, it can also bind to amino group derivatives in proteins, crosslinking with important digestive enzymes as well as dietary starch and proteins. This crosslinking further interferes substantially with the functionality and biological availability of the macromolecules [8,38].

The concentration of IP6 varies widely in raw BGN and AYB depending on the seed accessions and genetic differences. The phytate content of a sample of BGN flour was observed to be extremely high at 1470.15 mg/100 g, while another study observed the range to be from 1110 to 1511 mg/100 g; AYB: 133–501 mg/100 g [2,39]. Unigwe et al. [16] observed a range of 140 to 490 mg/100 g when 30 BGN accessions were analysed for phytate. A similar study of 90 accessions of BGN seeds obtained from the genetic resource centre of the IITA (Nigeria) revealed the same variation in the phytate concentrations, with the brown variety reported to contain 781.23 mg/100 g and the red variety containing 587.86 mg/100 g [16,43,44].

The variation in the concentrations of phytate is narrower in AYB seeds or flour when compared to the extensive range observed in BGN. The observed concentration in the raw AYB seeds is 501.61 mg/100 g phytate. Another study reported a concentration of 133 mg/100 g in the raw AYB flour [45]. Phytate is mostly concentrated in the seed coats of AYB. This is shown to be approximately 4.94 g/100 g, whereas the concentrations in the unprocessed raw AYB ranged from 3.4 to 8.1 g/100 g, depending on the accessions [31].

Another important antinutrient found in both BGN and AYB is tannin. Tannin is a water-soluble, complex biomolecule of polyphenol. The condensed form of tannins, such as proanthocyanidins, is the most abundant type, largely concentrated in the seed coat or testa of BGN and AYB. Tannin exerts its effect by directly binding strongly and irreversibly to dietary proteins through hydrogen bonding and hydrophobic interactions, leading to the precipitation of proteins [38]. The effect of the binding is that digestion of protein is greatly restricted, as well as the subsequent absorption of amino acids. Tannins also inhibit the activities of some digestive enzymes (e.g., trypsin, chymotrypsin and lipase), further reducing the nutritional quality and digestibility of protein. Just like phytates, tannins can also chelate cations like Fe^2+^ and Zn^2+^, thus reducing the bioavailability and absorption of these legumes.

The concentrations of tannins in BGN are observed to be associated with the seed colour, although this is not absolute. The darker-coloured seeds, such as black and red, are often observed to have higher concentrations due to genetic varieties. The observed concentrations in BGN range from 20 mg/100 g to 62 mg/100 g [16]. A study by Olanrewaju et al. [46] recorded a range from 1.1 mg/100 g to as high as 18.61 mg/100 g in BGN seeds. The reported content in 95 accessions ranged from 0.08% to 0.42% (or 80–420 mg/100 g). For AYB, the concentration of tannin is typically between 0.66 and 3.88% (660–3880 mg/100 g), with the highest concentration in the raw seeds [31]. There is a significant decline in tannins (as well as other antinutrients) during processing, such as fermentation, roasting, soaking and germination. In another study, fermented AYB showed a concentration of 0.99–0.50 g/100 g, and the concentrations in oven-dried samples ranged from 0 to 2.45% [45].

Other ANFs in these legumes include oxalates and trypsin inhibitors (TIs). While TIs act as serine-protease inhibitors, oxalates chelate minerals to form insoluble salts. The mechanism of action of TIs involves strong competition with dietary protein for the binding sites of the digestive enzymes. This activity of TIs inhibits the enzymatic activity of trypsin and chymotrypsin. This impedes the digestion of proteins in the gastrointestinal tract, resulting in lower digestibility of dietary protein. TI activity in BGN showed some variations, with a range of 5.3 to 73.4 TIU/mg in a study on 30 accessions [16]. In another study, the TI activity of raw AYB flour was observed to be 3.24 TIU/mg [31,45].

Oxalates, on the other hand, occur as either soluble salts (sodium or potassium oxalates) or insoluble salts (calcium oxalate). They reduce the bioavailability of nutrients by binding to divalent mineral cations such as Ca^2+^, Mg^2+^, Zn^2+^, and Fe^2+^, forming highly insoluble salts such as calcium oxalate. The formation of these insoluble complexes prevents the absorption of dietary Ca, thus interfering with its metabolism. The consumption of oxalates in high concentrations is linked not just to nutritional deficiencies in these minerals but also to the formation of kidney stones. The concentration of oxalates varies widely from the observations of different studies. For instance, raw BGN is reported to contain 6.14 mg/100 g, while the raw flour contains about 0.17–1.85 mg/100 g [20,47]. For the raw BGN seeds, the concentrations vary in different varieties from 2.43 mg/100 g, 2.80 mg/100 g and 2.47 mg/100 g in brown, cream and red varieties, respectively [48]. Dehulling, fermentation, and cooking are processing techniques that have been applied to reduce the concentrations of oxalates in BGN. The concentration in raw AYB is 2.25 mg/100 g, but decreased significantly by 80.36% (0.44 mg/100 g) when subjected to soaking and germination before incorporation into flour for complementary foods [48]. For both BGN and AYB, the concentrations of ANFs are reduced through processing such as roasting, soaking, germination, boiling, and fermentation. This reduction potentially mitigates antagonistic effects on protein digestibility and mineral bioaccessibility, thus making the products safer and more nutritious, although exact residual concentrations vary with processing conditions, accession, and analytical method used [33].

### 3.2. Fermentation as a Strategy to Enhance Nutrient Bioavailability

BGN and AYB are integral parts of most households in SSA, dating back millennia. This is because they require little or no input during cultivation, and can provide sustenance for people, as well as nutritional diversity, most especially during scarcity [3]. The preparation methods used traditionally are geared towards the need to overcome the hard-to-cook nature of these legumes, such as soaking, boiling, milling and roasting of the immature BGN seeds for a softer texture [6]. These approaches not only soften the seeds but also reduce the concentration of some ANFs.

Historically, spontaneous fermentation has been a vital preservation tool as well as a means of enhancing nutritional quality through the utilisation of microbial prowess to break down complex macromolecules and toxic compounds. Controlled fermentation, using diverse types of microbes, is explored in the contemporary context for these legumes in their applications as functional foods or ingredients. Controlled fermentation of BGN and AYB flours is utilised in the contemporary context, generating bioactive compounds such as γ-aminobutyric acid (GABA) and enhancing antioxidant activity and release of bound phenolics [49]. This approach bridges indigenous knowledge of fermentation with modern food biotechnology using standardised microbial cultures and optimisation parameters for consistent functional product and quality. Fermented BGN and AYB flours have been blended or used in combination with other cereals such as wheat or maize. For BGN, it has been incorporated into gluten-free bakery products aimed at consumers prone to celiac disease or intolerant to gluten, while at the same time serving as a source of the essential amino acid lysine, thereby enhancing the overall richness in nutrients and flavour complexity of the composite breads or biscuits [22,50]. This is specifically interesting for emerging markets in Africa and beyond, where the emerging trend is that consumers are demanding convenient plant-based (protein) alternatives.

Alkaline fermentation of legumes to produce condiments such as *dawadawa* is an age-long practice that has been carried out in many countries in West and Central Africa. The fermentation process reduces the beany flavour, imparts flavour and enhances “*meatiness*” in the soups and sauces to which the condiment is applied. Fermentation of BGN and AYB follows a solid-state alkaline fermentation analogous to the traditional production of *dawadawa* from African locust bean (*Parkia biglobosa*) but adapted to these underutilised legumes. This process is normally spontaneous, relying solely on naturally occurring microbes usually present on the food substrate. The type of fermentation is usually done at the household level using simple equipment, unlike controlled fermentation, which may require a bioreactor. The preparation of *dawadawa*, for instance, involves several crucial steps before the actual fermentation step from the raw legume seeds (such as African locust bean). These steps involve soaking, boiling (about 12 h for the preparation of African locust bean), dehulling, another round of cooking with some addition of potash, and incubation (involves wrapping with leaves/jute bags). These steps are then followed by fermentation for about three to five days, which occurs at ambient temperature [51].

The microflora of these traditional condiments consists of both spoilage and beneficial microbes. Some of these microbes are the result of contamination that occurs due to the actions of food handlers and equipment. The fermentation process of these legumes, just like the others, is predominantly driven by bacteria, with yeasts playing a less important role. The dominant microbial actors are members of the *Bacillus* spp. constituting more than 95% of the total microbial population density. This dominance may be attributed to their ability to form resistant spores capable of surviving many environmental stresses, including the initial boiling stages. This dominance of *Bacillus* is supported by a study where spontaneously fermented BGN dawadawa was found to consist of more than 95% of these spore formers [51]. However, some factors, such as substrate (BGN vs. locust bean or AYB), geographic region, process parameters (duration of boiling, potash addition to increase pH), and incubation temperature, potentially inhibit the growth of other microbes, such as *Staphylococcus*, *Enterococcus*, or LABs, in the early phase of fermentation.

Some of the species of *Bacillus* commonly associated with BGN fermentation are *B. subtilis*, *B. licheniformis*, *B. pumilus*, *B. cereus*, *B. firmus*, *and B. amyloliquefaciens. B. licheniformis* has been observed to be the central player in the fermentation of BGN into a *dawadawa*-type condiment [48,49]. The risk of the pathogenic *B. cereus* can be mitigated by the use of non-toxigenic starter cultures coupled with strict hygienic practices. This microflora is similar to that of the AYB, especially in the production of *owoh* [52]. There are limited studies on the fermentation of AYB [51]. The primary physiological function of *Bacillus* (for protease activity) is to hydrolyse proteins into peptides, amino acids, and ammonia, creating the unique alkaline pH (8.5–9). This high-pH environment suppresses the growth of spoilage microbes as well as some potential pathogens. Other bacteria associated with the spontaneous fermentation of BGN are *Lysinibacillus fusiformis* LMG 18,474, *Phyllobacterium leguminum* ORS1419, *Staphylococcus saprophyticus*, and *Streptococcus* spp. Some fungi and moulds, such as *Aspergillus niger*, *Penicillium* spp., *Rhizopus* spp., and *Saccharomyces cerevisiae*, are among the natural starters during spontaneous fermentation of BGN into traditional condiments [51,52]. Lactic acid bacteria (LABs) are also identified with this type of fermentation, especially when BGN is fermented with grains such as maize in the production of plant-based yoghurt and other beverages. Examples of such LABs are *Leuconostoc*, *Lactiplantibacillus*, *Streptococcus*, and *Pediococcus* species [53]. LABs have been equally used as starter culture in the production of certain unique products from BGN under controlled conditions. Such LAB starter cultures include *Lactobacillus delbrueckii* subsp. *bulgaricus*, in combination with *Streptococcus thermophilus*, which is used as a starter culture in the controlled fermentation of Bambara groundnut milk into plant-based yoghurt [24]. One of the disadvantages of spontaneous fermentation, when compared with controlled, besides product inconsistency, is that the outcome is a mixed microbial population consisting of the active fermenters, spoilage and pathogens such as *B. cereus* and aflatoxin-producing *Aspergillus flavus*. Of these two pathogens, the toxigenic strains of *B. cereus* are the most prominent, constituting about 35% of isolates during the spontaneous fermentation of BGN *dawadawa*. *B. cereus* produces two toxins, which are the heat-stable emetic toxin cereulide and the heat-labile diarrheal enterotoxins (Hbl, Nhe, CytK). These toxins cause rapid-onset vomiting, abdominal cramps and watery diarrhoea 6–15 h after ingestion. Table 2 gives the detailed impacts of fermentation and other pre-treatment steps on nutrient bioavailability and degradation of ANFs in these legumes. The reported reductions in ANFs, as shown in the table, are characteristically the result of multi-step protocols which include soaking, germination, dehulling, or thermal pre-treatments. These pre-treatments contribute to the breakdown or leaching of ANFs with fermentation further amplifying the process of degradation through enzymatic activity. The table should be interpreted holistically rather than attributing the degradation solely to the action of fermentation.

Controlled fermentation involves the deliberate introduction of well-defined starters, often molecularly typed, into the fermentation system in such a way that only the microbes of choice are favoured to grow. This helps to standardise the process, providing solutions to the challenge of product inconsistency and safety issues linked with the spontaneous fermentation process. The process may involve the use of optimised process parameters, such as inoculum size, while ensuring the conditions are sterile. Some typed strains have been used as starters in the production of *dawadawa*-like condiments produced from BGN. Examples of such starter cultures are *B. subtilis* subsp. *subtilis* SFBA3, *B. amyloliquefaciens* subsp. *plantarum* SFBA2, and *B. licheniformis* OALB2. Others are LABs such as *Lactobacillus delbrueckii* subsp. *Bulgaricus*, in combination with *Streptococcus thermophilus* [8,51]. Unlike spontaneous fermentation, controlled fermentation offers several advantages, including product consistency, enhanced product quality, and improved safety.

### 3.3. Degradation of ANFs by Microbial Enzymes

There are two classes of enzymes that play a role in the degradation of ANFs during the fermentation process: exogenous enzymes, usually secreted by the fermenting microbes, and endogenous enzymes present in the seeds, with microbial enzymes playing a greater role. For phytate, the reduction is mainly attributed to phytases (phosphoric monoester hydrolases, E.C. 3.1.3). Phytases hydrolyse phytate into lower inositol phosphates, such as IP5, IP4, and IP3, and sometimes free myoinositol. This degradation of the phytates into the lower forms with reduced affinity for minerals, when compared with IP6, decreases their inhibitory effect on mineral absorption [7]. Many species of microbes, such as *Bacillus* spp., *Saccharomyces cerevisiae*, and *Lactobacillus* spp., either secrete phytases or stimulate the activity of endogenous phytase within the seeds of the legumes. Lactic acid fermentation influences the activity of phytase through the low pH and stabilisation of the enzyme [54]. The mechanism of phytate reduction differs between alkaline and lactic acid fermentation. Phytate reduction mechanisms differ markedly between fermentation types. During LAB fermentations, the breakdown of phytate occurs mainly through acidification (pH drops to ~4–5.5). The drop in pH activates plant endogenous phytases with little contribution from the microbial phytase. This is not the same during alkaline fermentations by *Bacillus* spp. in which the degradation of phytates relies mainly on microbial phytases. The process contributes to effective phytate breakdown alongside proteolysis and other biotransformations.

Fermentation is reliably documented as an effective means of reducing phytates in BGN. Several studies have revealed that fermentation generally reduces the concentration of phytates by approximately 18.1% to 95.9% [8,54]. This reduction is largely associated with certain fermented condiments, while higher concentrations are observed in treated flours. For instance, in BGN used for *dawadawa*, phytate was observed to have reduced within the range 18.1% to 25.6%. In another instance that utilised solid-state fermentation (SSF) using fungi as starters, the reduction was recorded to be up to 70% [46]. Another study in BGN flour using SSF and *Rhizopus* species as starters observed a broader reduction between 39.4% and 95.9% [45,48]. Different studies also combined pre-processing steps such as boiling (15 min–1 h at 100 °C), dehulling (manual/mechanical) and soaking (24 h at ~24–30 °C) before the controlled fermentation of the BGN seeds (30–45 °C for 84–120 h) with or without inoculum (usually 10^6^–10^8^ CFU/g substrate). One such study used cooking as a treatment step before the controlled fermentation using *Lactobacillus plantarum* and *Lactobacillus fermentum*, and observed a reduction from 1470.15 mg/100 g in the raw BGN seeds to 1023.10 mg/100 g, which is approximately a 30.41% reduction. Another similar study using BGN flour and *Saccharomyces cerevisiae* was able to reduce the time of fermentation to 48 hr and attained a 54.55% reduction in the concentration of phytate [20,55]. A similar trend of substantial reduction (of up to 39.85%) in the concentrations of phytate was observed in AYB flour when SSF was used for the fermentation process alongside *Saccharomyces cerevisiae* as a starter culture. The fermentation may be confounded by soaking/germination/thermal steps. Information about fermenting AYB only, without other pre-treatment steps (e.g., soaking, germination, etc.), is limited. Nonetheless, processing steps, such as soaking and germination, have been observed to reduce phytates by 82.01% [45]. Table 2 gives the detailed impact of fermentation and other pre-treatment steps on nutrient bioavailability and degradation of the antinutrients present in BGN and AYB.

#### Degradation of Tannins, Phenols and Proteinaceous ANFs

The breakdown of tannins during fermentation of BGN and AYB depends on the tannin subclass, substrate and the fermenting microbes. The subclasses are hydrolysable tannins (HT) and condensed tannins (CT). HTs are susceptible to microbial tannases and esterases, especially from LABs. HT consists of gallic or ellagic acid esters linked to a polyol core. This results in their depolymerisation into simpler, less astringent phenolic compounds such as gallic acid, leading to significant reductions (between 50 and 90%) [50,56]. CTs, such as proanthocyanidins and flavan-3-ol oligomers/polymers, are less prone to microbial breakdown because they are more structurally resistant. They are usually found in the seed coat of darker varieties and may be leached partly during dehulling and soaking pre-treatments. Microbial tannases (tannin acyl hydrolase, E.C. 3.1.1.20) or polyphenol oxidase (PPO) enzymes are primarily responsible for the decline of tannins during the fermentation. They exert their activities by hydrolysing the ester and depside bonds present in tannins [53]. PPO also acts on tannins by polymerising the compound, hence inactivating it. This hydrolytic action leads to a reduction in the concentrations of these antinutrients, thus enhancing nutrient bioaccessibility [12].

Proteolytic enzymes readily break down the proteinaceous ANFs, possibly into inactive forms. LABs have exceptional proteolytic systems involving proteinases bound to the cell wall, peptide transporters, and numerous intracellular peptidases that hydrolyse protease inhibitors such as TIs, reducing their inhibitory activity in the process. Lectins are also degraded by microbial and endogenous enzymes, as well as pre-treatment, causing the disruption of the structure and reducing hemagglutinating activity. Though the thiol exchange involving glutathione, which disrupts the disulphide bonds, has been implicated, there is limited evidence of its mechanism [48]. The total degradation or reduction of these antinutrients is very important for the improvement of the nutritional values and bioavailability of these legumes. Besides these enhancements of nutritional value and bioavailability, fermentation also significantly increases in vitro protein digestibility and the availability of amino acids (AAs). While alkaline fermentation releases ammonia through transamination, thus elevating the pH (8.0–9.5) and also contributing to the characteristic flavour, acidic fermentation enhances the production of metabolites such as lactic and acetic acids. Some of these AAs are by-products of proteolytic action of the enzymes from the release of proteins that are otherwise bound to certain antinutrients [8,55].

### 3.4. Production of Peptides, Phenolic Compounds, and Other Metabolites with Potential Health Benefits

Fermentation is a strong bioprocessing tool proven to significantly transform BGN and AYB, leading to the production of several novel compounds, phenolics and bioactive peptides with functional health benefits such as potential antioxidant or antihypertensive properties. The release of bioactive peptides is common during alkaline fermentation as a result of the different actions of microbial proteases on the substrates. For instance, during the fermentation of AYB with some monoculture bacterial starters, the resulting condiments were observed to possess heightened nutritive values and higher levels of amino acids [19]. Studies on protein hydrolysates of fermented BGN also revealed several peptide fractions with health-promoting properties. The potential use cases include the management of oxidative-stress-related metabolic disorders, in vitro inhibition of angiotensin-converting enzymes (ACEs) and renin, indicating a potential cardio-protecting effect by lessening blood pressure [27].

Besides their impact on the bioactives, fermentation also increases the total phenolic content (TPC) of AYB flour. Specifically, fermented AYB flours were observed to have higher concentrations of TPC (0.47 mg/g) when compared to unfermented (0.33 mg/g), with some increases as high as 80% [3]. This increase is linked with the degradation of the cell wall contents in the legumes, as well as ANFs and high-molecular-weight polyphenols. The enzymatic actions of fermenting microbes also contribute by releasing bound phenolics and synthesising new ones, adding to the overall increase in TPC. The richness in phenolics and flavonoids contributes immensely to their antioxidant properties when consumed, positioning the fermented products as functional foods.

The same increase in the concentrations of phytochemicals and bioavailability was also observed during the fermentation of BGN into condiments like *dawadawa.* Raw BGN is equally rich in phenolic acids (derivatives of caffeic acid, quinic acid), flavanols (catechin, epicatechin) and flavonols (quercetin, kaempferol, rutin, myricetin), which are known for their antitumour, cardioprotective, antimicrobial and antioxidant properties [27]. Generally, there is a decrease in the concentration of phenolics during the fermentation of BGN, such as medioresinol, quercetin-3-O-galactoside-7-O-rhamnoside and quinic acid. Despite this, there is an overall increase in total antioxidant activity. *Dawadawa* condiments produced from hulled BGN are observed to have higher concentrations of phenolic composition, suggesting vital health benefits. Alkaloid and flavonoid derivatives (e.g., alkylphenones, iridoids), known for their pharmacological and antimicrobial properties, are easily found in BGN *dawadawa* [44].

#### Comparison of Metabolite Profiles in Fermented BGN and AYB

BGN fermented with *Bacillus* spp. into the condiment *dawadawa* typically exhibits a complex profile of volatiles and non-volatiles. Some of these metabolites include ketones, aldehydes, sterols, alcohols, nitrogen-containing compounds, furans, pyridines, acids, vitamins, fatty acids, sulphur-related compounds, esters, terpenes, terpenoids, and esters [46]. The esters (37%) dominate the volatiles, contributing greatly to the unique aroma of the condiments. During the fermentation of the dehulled BGN, specific ester derivatives observed include palmitic acid, ethyl ester (17.7% peak area), lauric acid, ethyl ester (10.2% peak area), 9,12-octadecadienoic acid (Z, Z), and 2-hydroxy-1-(hydroxymethyl)ethyl. The formation of esters arises through the esterification of alcohols and fatty acids or through the acid produced by microbes (such as LABs) and alcoholic metabolites. Besides the formation of the esters, the fermentation may lead to the modification of essential fatty acids (EFAs) such as oleic, linoleic, and linolenic acids, further enhancing the extractability of the product and enabling it to reduce the risk of coronary heart disease and lower blood cholesterol and pressure [24,46].

Specific strains of the fermenting microbe are also linked to the production of unique metabolites. A good example of this is *B. subtilis*, *which* produced acetic acid and hexadecanoic acid [50]. Other compounds, such as ketones, 2,5–dimethyl pyrazine and alcohol, were observed in the product fermented by *B. cereus*, with the primary ketone as 2-Butanone (methyl ethyl ketone). Hexanal was observed for the BGN fermented by *B. licheniformis*, while alcohols, aldehydes and acids were over 70% of the volatiles fermented by *Bacillus* spp. Other major metabolites, such as *ϵ*-tocopherol (vitamin E), were identified in dehulled BGN fermented *dawadawa* samples. For the fermented AYB condiment, there was also a noticeable increase in the concentrations of the acidic amino acid, aromatic amino acids and total essential amino acid underpinning the proteolytic degradation of proteins into more bioavailable forms [33,51]. In addition, the TPC also significantly increased from 1.19 mg GAE/g to 2.35 mg GAE/g AYB, signifying the effects of enzymatic breakdown and release of the bound forms of the metabolites. The effects of fermentation on the fatty acid content are not clearly known. Essentially, fermentation enhances the functional and nutritional profile of both BGN and AYB, resulting in a complex mixture of metabolites [34].

### 3.5. Impacts of Fermentation Techniques on Nutrient Bioavailability and ANF Degradation

The techniques applied to the fermentation of these legumes have some effects on the products, as shown in Table 3. These techniques differ in the aspects of microbial control and the physical state of the substrate being fermented, which may influence the outcome or product. Some of these techniques include spontaneous fermentation (SF), controlled fermentation (CF) or induced fermentation, solid-state fermentation (SSF) and submerged fermentation (SmF). SF is used in the traditional setting, relying solely on the natural microbiota on the legumes or in the environment. It is commonly used in the production of *dawadawa* [8]. In contrast with CF, SF is largely unpredictable because the microbes, as well as the inoculum size, are unknown, resulting in product variations and safety concerns. CF, on the other hand, applies defined or characterised starters or starter cultures carefully selected for the certain unique characteristics they impart to the fermentation system [53]. Some of the starters used are *B. subtilis* subsp. *subtilis* SFBA3 (good for the production of flavour compounds such as dimethyl disulphide), *B. amyloliquefaciens* subsp. *plantarum* SFBA2, *B. cereus* PALB7, and *B. licheniformis* OALB2, and for the fermentation of AYB, *B. amyloliquefacien*, *B. subtilis* and *B. siamensis* [19,50]. Substantial reduction in ANFs (e.g., oxalates) and aflatoxins (e.g., AFB_1_, AFG_1_, fumonisins) has been achieved when CF is used [57]. For BGN flour, phytate was reduced from 39.4 to 95.9% and TI from 37.3 to 87.1%, respectively. One of the limitations of CF is the expense in procuring the starters, which is normally out of reach for the artisanal fermentation of these legumes [8]. Empirically, SF increases mineral bioaccessibility and in vitro protein digestibility (IVPD) in BGN from 70.74% to 89.70% post-fermentation. Besides this, flavour profiles, bioavailability, crude protein (decreases by cooking step) and vitamins are enhanced significantly, with the concentrations of riboflavin and niacin increasing by two- and seven-fold, respectively [55]. When this approach is combined with other process parameters, such as temperature and time of fermentation, it provides greater control while at the same time ensuring reliable and reproducible outcomes with enhanced safety and consistent product quality.

Another way of differentiating the fermentation technique is by using the physical state of the legume or substrate being fermented. SSF makes use of solid substrates with reduced surface, such as flour, to maximise the contact for microbial action [8,16]. For example, studies using African yam bean (AYB) found that preparing the flour first and then subjecting it to SSF reduced the substrate through milling for effective microbial action. Conversely, the SmF approach (or liquid-state fermentation), just as the name suggests, involves the suspension of the legume seeds or substrate in an aqueous medium. This allows for the fermenting microbes to adhere to the seed surface before the inward penetration of the microbes through enzymatic degradation. Comparative analysis of previous studies suggests that SmF may be better for producing certain metabolites, e.g., antihypertensive compounds and GABA. On the other hand, SSF may produce a greater amount of soluble phenolic compounds and antioxidant activity. Irrespective of the physical state of the fermenting system, optimal fermentation usually needs consideration of some extrinsic and intrinsic parameters [34]. Table 3 shows a comparison of the impacts of different fermentation techniques on the nutrient bioavailability of BGN and AYB.

The commonly used starters for SSF are fungi such as *Rhizopus*, *Aspergillus*, and *Saccharomyces*. *Saccharomyces cerevisiae* has been used in the SSF of AYB flour, with an observed reduction in % ANF reduction [45]. The observed reductions are as follows: trypsin inhibitor by 58.02%, phytate by 39.85%, and tannin by 21.43%. For BGN products generally (including those derived from SSF), TI is reduced by 38.3–40.9%, phytate by 18.1–25.6%, oxalate by 26.6–59.1%, and tannin by 34.2–76.4%. SSF also leads to a noticeable increase in crude fibre and essential minerals, such as Fe and Zn, as well as enhanced bioavailability of phenolics. The increased TPC was observed to be 80.3% in fermented AYB flour because bound phenolic compounds were released, while degradation of phytates was linked to the increased mineral content. The reduced fat content observed after fermentation is desirable, as it can lead to longer product storage life due to minimal lipid oxidation. The limitation of SSF is that it can lead to a decrease in soluble fibres as a result of enzymatic hydrolysis [8,45].

For SmF, LABs are often used; for example, in the fermentation of AYB, tannins, alkaloids, and other ANFs decreased significantly as compared to unfermented AYB flour. This has also resulted in higher concentrations of crude protein values by up to 23.30%, and some minerals, especially K and P [45]. The mechanical effect of the process, which reduces particle size and disrupts the food matrix, is suggested as a reason for the increased protein [34]. This makes SmF a vital technique for the production of flours with high protein contents compared to SSF, with a consequential decrease in crude fat. SSF adopt the use of low water content (moisture typically 30–80%, with the microbes bound on the solid substrates.

The technique is generally used in the production of many African condiments such as *iru*, *ogirri* and *dawadawa*. The dominant fermenting microbes are sporulating microbes such as *Bacillus*. Other bacterial species playing less important roles in the BGN *dawadawa* process are *Staphylococcus saprophyticus*, *Streptococcus* spp., and some fungi such as *Saccharomyces*, *Rhizopus*, *Aspergillus*, and *Penicillium* species. The observed % reduction in ANFs during the fermentation of BGN for TI activity is 38.3–40.9%, phytate 18.1–25.6%, tannin 34.2–76.4%, and oxalate 26.6–59.1%, respectively. The degradation of ANFs may be heightened for BGN flour when fermentation (with *L. plantarum* and *L. fermentum*) is combined with other treatments, such as cooking. This was recorded as 4.72→2.08 mg/100 g for tannins, 1470.15→1023.10 mg/100 g for phytates, and 8.40→3.30 mg/100 g for TI activity, respectively [8].

### 3.6. Applications and Implications

Fermented BGN and AYB food products and ingredients show an incredible potential for combating nutritional challenges, specifically deficiencies in micronutrients and diseases linked to diets. Some fermented (legume) condiments were observed to attenuate oxidative stress and hepatic damage in streptozotocin-induced diabetes in rats. Such observations positioned fermented BGN and AYB as model legumes to be utilised in the development of functional foods, nutraceuticals and probiotic beverages [27,58]. Traditionally, these legumes can find some uses in the household setting in SSA targeted at improving rural food security. BGN and AYB can readily be processed into *dawadawa*-like condiments, simple fermented flour/porridge, or as BGN milk using conventional house equipment. Probioticated BGN milk not only has a comparable nutritional composition to soybean milk, but better consumer acceptability than soybean and cowpea milk, thus contributing to food diversity, security and sustainability. Since BGN and AYB are nutrient-dense, they are potentially important in the development of complementary foods and fortified food products targeted at mitigating protein-energy malnutrition (PEM) and micronutrient deficiencies prevalent in low–middle-income countries (LMICs) [8]. On an industrial scale, BGN and AYB can serve as inexpensive fortifiers for the improvement of less nutritious staples like maize and sorghum, enhancing their overall nutritional value. Several studies reinforce this assertion that BGN-enriched complementary foods, either used as a stand-alone or in combination with other ingredients like maize, popcorn, and African locust bean, revealed enhanced protein content, energy values, and balanced essential amino acid profiles, either comparable or superior to commercial infant formulas, thus satisfying nutritional guidelines for infants [3].

Any of the fermentation techniques can be optimised and scaled up to produce value-added products from BGN and AYB, providing industrial and commercial opportunities. These value-added products may include flours, while at the same time improving the techno-functional properties of the products [58]. Fermented BGN and sorghum flours, for instance, are identified as useful ingredients for commercial and industrial purposes. The development of such products needs to be concurrent with their sensory analysis to determine consumer acceptability and market potential. Some of the challenges linked with these legumes, especially BGN, include the persistent “*beany flavour*” and lack of familiarity among consumers [8]. Consequently, optimisation of the processing parameters and techniques is required to enhance sensory appeal and create a wider market adoption. Fermented BGN and AYB can be utilised in various forms (e.g., flour, extruded snacks, milk, yoghurts, condensed products, complementary foods, condiments), thus providing extensive industrial applicability. In the long run, the commercialisation of underutilised crops, specifically through industrial valorisation, will foster sustainable food security, while at the same time boosting the financial status of the smallholder farmers and elevating the global status of these climate-resilient crops [11,21].

## 4. Research Gaps and Future Directions

Despite the promising roles of fermentation as a tool for improving the utilisation of BGN and AYB, there are still several key gaps limiting the translation of available research into useful applications. This implies that future research must emphasise robust and standardised experimental and validation pipelines that transcend the usual compositional analyses to efforts geared towards assessing bioavailability, efficacy and safety of the products.

Notable among the existing gaps is the obscure contributions to the reduction in ANFs and nutrient enhancement because of the confounding roles of fermentation that can be attributed to the heterogeneity of fermentation protocols (such as spontaneous vs. controlled) and pre-treatment steps (dehulling, soaking and germination). Priority must be given to experimental designs targeted at a single process, such as in vitro digestion models, rather than in vivo. This must be combined with assays such as mineral dialysability, useful for quantifying the bioaccessibility of important micronutrients. This will promote the reproducibility of data and also reveal the roles of fermentation in the release and solubilisation of minerals under simulated digestion. Since the reduction in phytate dominates most studies, the implication for the absorption of minerals needs to be explored. Future studies need to integrate phytate:mineral molar ratio calculations pre- and post-fermentation, incorporated with a tissue culture (Caco-2 cells) assay to determine transport and permeability of minerals/phenolic fractions. The limited *in vivo* data, specifically in the case of AYB, presents an obstacle to evidence-based recommendations. This challenge can be addressed by using an animal (rodent) model to measure unique biomarkers such as plasma ferritin, tissue zinc level, etc., for the assessment of long-term bioavailability and health outcomes, such as the prevention of anaemia. This can then be followed by human intervention studies focused on at-risk populations using strategies such as complementary foods, etc., and clinical assessment of certain changes, such as cardioprotective claims.

**Figure 1:**
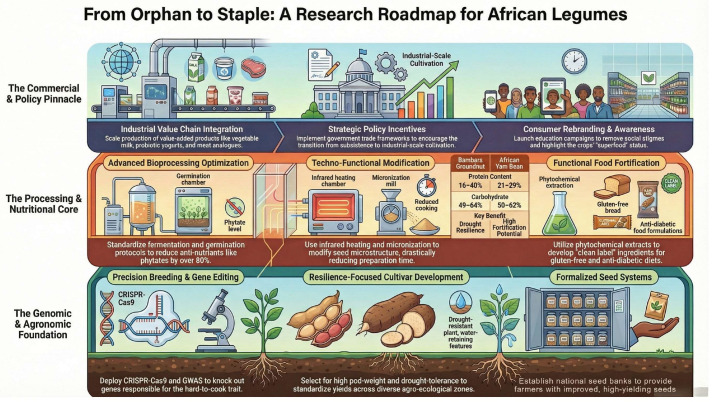
Proposed research roadmap to address gaps and enhance the nutritional and commercial potential of Bambara groundnut and African yam bean (generated using NotebookLM).

It is also important to incorporate pipeline validation targeted at the mycotoxin reduction claims during the fermentation. This implies the screening of the raw seeds for certain mycotoxins (such as aflatoxins/fumonisin, etc.) using HPLC-MS. This can then be followed by controlled fermentation with constant monitoring of the levels of toxins and assessment of the microbial dynamics using molecular tools such as qPCR targeted at toxin-producing microbes like B. cereus, Aspergillus and genes responding to the degradation of these toxins. The adoption of these pipelines has the potential to bridge the knowledge gaps and provide evidence-based justification for the adoption of fermented products from BGN and AYB. Figure 1 proposes a roadmap addressing the existing gaps for the adoption of these legumes at both the household and commercial levels.

## 5. Conclusions

This review highlights the significance of fermentation as a beneficial bioprocessing strategy for potentially improving the bioavailability of essential nutrients, although empirical evidence from in vivo studies remains unconfirmed in the current literature. This reduction depends on the antinutrient, substrate, microbial strains, fermentation type, and pre-treatment step (the reductions are highly process- and substrate-dependent). Although fermentation is relatively cost-effective and requires low energy input, it offers a viable solution to valorise indigenous crops while enhancing the nutritional quality and functionality of these crops. This has the potential of positioning BGN and AYB as vital tools in mitigating the challenges of protein-energy malnutrition (PEM) and micronutrient deficiencies, particularly among vulnerable populations in developing countries. The versatility of these climate-smart, underutilised legumes enables their effective use in traditional dishes, enrichment of staple foods, and the formulation of affordable food products. Despite this promising prospect, the full nutritional and commercial potential of these legumes may be hindered unless there is a deliberate and concerted effort geared towards research and innovation in the development of nutritious complementary foods. Important technical obstacles to their adoption (e.g., hard-to-cook (HTC) phenomenon, undesirable beany flavour) must be scientifically addressed.

We acknowledge several limitations across the studies used. Such limitations include the heterogeneity in the methods of fermentation, pre-treatment steps, microbial strains, and genetic variations of the legumes, which may hinder comparisons of the outcomes and generalizability of outcomes. Most of the data depends on in vitro proxies rather than in vivo studies on human subjects. Also, studies focusing on controlled fermentation of AYB and the long-term outcomes of such products are specifically scarce when compared with BGN. Such gaps highlight the need for standardised protocols, in vivo validation, and more robust studies on AYB to fully realise the potential of the crop in food security and public health strategies.

## Figures and Tables

**Table 1:** Nutritional composition and antinutrients present in Bambara groundnut and African yam bean compared to the mainstream or common legumes expressed in mg/100 g dry weight.

Nutrient/ANF	Bambara Groundnut (Range)	African Yam Bean (Range)	Mainstream Legumes (e.g., Soybean, Cowpea)	Impact on Bioavailability	References
Protein	9.6–40.0% (typically 15–28%)	19.0–30.0%	Cowpea: 19.00–27.00%; Soybean: up to 36%	Proteins are essential nutrients. High concentrations of antinutritional factors (ANFs) reduce protein bioavailability and digestibility. Processing methods such as cooking and fermentation increase protein digestibility.	[22]
Carbohydrate	42.0–75.30% (largely starch and neutral detergent fibre)	49.88–67.36% (carbohydrate and protein are major components)	Cowpea: 56.00–64.00%; Soyabean: 17.58–22.47% (in specific genotypes, can be as high as 52.74%)	Carbohydrates provide a source of energy. ANFs can impede the digestibility and absorption of carbohydrates. BGN has a higher proportion of slowly digestible starch and resistant starch in the raw form.	[37,38]
Fat/Lipid	4.30–12% (low, typically < 10%)	0.37–7.53% (low content is ideal for weight management)	Cowpea: 1.00–4.50%; Soybean: 20–28.2%	BGN fat is mostly unsaturated (oleic and linoleic acids), beneficial for health.	[11,37,39]
Fibre (Crude/Dietary)	1.4–12.9% (crude/dietary fibre; insoluble fibre generally higher than soluble)	2.47–9.57% (crude fibre)	Cowpea: 1.30–2.50%; Soybean: 9.3% dietary fibres	High fibre consumption lowers the incidence of lifestyle diseases. Excessive fibre may decrease appetite in infants. Fibre, along with other compounds, can synergistically inhibit mineral bioaccesibility.	[3,11,37]
Essential Amino Acids (EAAs)	Rich in lysine and contains relatively high methionine compared to other legumes; lysine 1.20–10.32% of protein; methionine 0.50–6.41% of protein	High amount of EAAs; profile comparable to a whole chicken egg	Legumes are often deficient in methionine, tryptophan, and cysteine, though rich in lysine; cereals are deficient in lysine	BGN is lysine-rich and methionine-poor, making it a good complementary protein source for cereals. ANFs (like tannins) interfere with amino acid bioavailability by forming irreversible complexes with protein.	[11]
Tannin	0.11–1861 mg/100 g (condensed tannins found mainly in the testa/seed coat, higher in darker seeds)	Raw seed value reported up to 713.50 mg/100 g; seed coat range: 2.06–6.59 mg/100 g	Common bean: 600–2840 mg/100 g; Peas: 2780–3090 mg/100 g	Reduces the digestibility and bioavailability of proteins and amino acids by forming indigestible complexes. Tannins can impart bitterness and astringency, which can affect palatability. Tannins in low concentration may offer beneficial effects (e.g., antioxidant activity, hinders internal parasites).	[6,40,41,42]
Phytate/Phytic Acid	110–1511 mg/100 g (PAP: 140–490 mg/100 g)	Raw seed range: 2.06–501.61 mg/100 g	Common bean: 60–80 mg/100 g; Peas: 120 mg/100 g; Soyabean: 16 to 707 mg/100 g of dry seed	Interferes with mineral absorption (Fe, Zn, Ca, Mg) by chelating to mineral cations to form stable, indigestible complexes. Phytic acid can also impair protein digestibility by crosslinking with dietary proteins and digestive enzymes. Also noted to have potential health benefits (antioxidant, anti-cancer properties).	[6,40,41,42]
Oxalate	Detected; seed coat range: 9–23 mg/100 g	Raw seed range: 0.18–2.25 mg/100 g	Kidney beans: 1.5–1.8 mg/100 g in raw samples; Soyabean: 670 mg to 3500 mg/100 g	Reduces mineral bioavailability, particularly calcium, by binding to metal cations.	[40,43]
Trypsin Inhibitor	6–7340 TI mg/100 g (trypsin inhibitor content ranges from 5.3 to 73.4 TI/mg)	Seed coat range: 18.96–27.45%	Kidney beans: 4040 mg/100 g; Soyabean: 1320 to 3393 mg/100 g and other legumes (protease inhibitors)	Impedes protein digestion and absorption by inhibiting protease activity. Low trypsin levels can cause pancreatic hypertrophy. Processing effectively reduces or eliminates this factor.	[6,42]

All values are expressed on a whole-seed, dry-weight basis unless otherwise specified. Seed coat values are typically higher due to the concentration of antinutrients in the test.

**Table 2:** The detailed impacts of fermentation and other pre-treatments (e.g., dehulling, soaking, milling, germination) on nutrient bioavailability and degradation of the antinutrients present in Bambara groundnut (BGN) and African yam bean (AYB).

Component/Nutrient Category	Bambara Groundnut (BGN) Range (mg/100 g Dry Weight)	African Yam Bean (AYB) Range (mg/100 g Dry Weight)	References
Phytates	**Reduced Substantially:** Raw BGN analysed at 617.01 mg/100 g, reduced to 108.92 mg/100 g after combined soaking and germination (**82.34% decrease**). In maize–BGN complementary food blends, phytate content dropped from 180 mg/100 g to 100 mg/100 g after fermentation and malting. Fermentation/germination/roasting reduces ANFs, including phytate.	**Reduced Substantially:** Raw AYB analysed at 501.61 mg/100 g, reduced to 90.23 mg/100 g after combined soaking and germination (**82.01% decrease**). The native AYB phytate content typically ranges from 3.18 mg/100 g (unprocessed, lowest value) to 5.86 mg/100 g (unprocessed, highest value).	[3,8,16,31]
Tannins	**Reduced Significantly:** Raw BG recorded 315.01 mg/100 g, reduced to 49.32 mg/100 g after processing (**84.34% decrease**). Tannin levels in Bambara complementary food blends were reduced to 150 mg/100 g upon fermentation.	**Reduced Significantly:** Raw AYB recorded 713.50 mg/100 g, reduced to 56.15 mg/100 g after processing (**92.13% decrease**). In one study, tannin content in submerged fermented AYB (FAYB) flour was reduced to 0.82 TAE/100 g compared to 1.00 TAE/100 g in the native sample. Unprocessed AYB tannin content varied widely (e.g., 0.66% to 3.88%), with processing significantly reducing levels, sometimes to 0.00% in certain accessions.	[8,31,34]
Alkaloids and Cyanic Acid	Processing (soaking/germination/drying) reduced **cyanic acid** content from 3.51 mg/100 g (raw) to 1.75 mg/100 g (**50.04% decrease**).	Processing (soaking/germination/drying) reduced **cyanic acid** content from 5.03 mg/100 g (raw) to 2.01 mg/100 g (**60.14% decrease**). **Alkaloid** content reduced from 2.90% (native AYB) to 2.28% in submerged fermented AYB (FAYB).	[34]
Oxalates	Processing (soaking/germination/drying) reduced oxalic acid content from 1.96 mg/100 g (raw) to 0.35 mg/100 g (**82.14% decrease**). Oxalate content in Bambara complementary food blends was reduced to 20 mg/100 g upon fermentation.	Processing (soaking/germination/drying) reduced oxalic acid content from 2.25 mg/100 g (raw) to 0.44 mg/100 g (**80.36% decrease**).	[53]
Crude Protein Content	**Increased:** Crude protein content significantly increased during alkaline fermentation (e.g., in *dawadawa* production). Raw BGN had 1873 mg/100 g protein, while dehulled *dawadawa* (fermented) had 2310 mg/100 g, and hulled *dawadawa* had 22.05 g/100 g. Fermentation also increased the protein percentage in BGN flour blended with sorghum (e.g., from 10.15% to 10.89% after 24 h of fermentation).	AYB seeds are protein-rich (19–30%). The crude protein increased from 22.81 to 24.00%, depending on the period of fermentation.	[3,20,46]
Amino Acid Profile	**Enhanced:** Fermentation (into *dawadawa*) resulted in increased concentrations of several essential amino acids (e.g., leucine, phenylalanine, and threonine) and non-essential amino acids (e.g., arginine, serine, glutamic acid, and alanine) compared to raw BGN. For example, glutamic acid (NAA) increased from 256 mg/100 g in the raw sample to 307 mg/100 g in dehulled dawadawa and 280 mg/100 g in hulled dawadawa. For essential amino acids like methionine, the observed increase was from 24 mg/100 g to 32 mg/100 g in the dehulled dawadawa and 26 mg/100 g in the hulled sample.	AYB protein contains an appreciable level of most essential amino acids. The total essential amino acids increased from 35,750 to 46,320 mg/100 g protein, while total non-essential amino acids increased from 38.29 to 49.47 g/100 g protein, depending on the fermenting bacteria.	[9]
Mineral Content & Bioavailability	**Increased/Improved Bioavailability:** Fermentation generally increases the ash content in BGN complementary foods (e.g 1950 mg/100 g to 3005 mg/100 g). Iron (Fe) 56%, potassium (K) 67.16%, magnesium (Mg) 24.24%, phosphorus (P) 12.82%, zinc (Zn) 16.71%.	**Enhanced/Increased Phosphorus: Phosphorus** content substantially increased from 26.54 mg/100 g (native AYB) to 56.23 mg/100 g (FAYB). Phosphorus (P) +111.87%, potassium (K) +4.96%, magnesium (Mg) −0.84%, calcium (Ca) −14.46%.	[3]
Total Phenolic Content (**increased TPC**)	The impact of fermentation on the TPC of BGN is more complex and depends on whether the concentration is measured in the whole product (including hulls) or the degree of reduction in specific polyphenols (like tannins).	**Increased by Fermentation:** Fermentation enhances the bioavailability of phenolic compounds by breaking down cell walls and hydrolysing antinutrients/high-molecular-weight polyphenols. Fermented AYB seeds demonstrated higher TPC (0.47 mg/g) than unfermented samples (0.33 mg/g). Submerged fermented AYB (FAYB) flour exhibited a TPC of 2.35 mg GAE/g, significantly higher than the native AYB control (1.19 mg GAE/g)—an 80.3% increase.	[3,8,42]
Total Flavonoids Content (TFC)	Raw BGN seed coat exhibited TFC ranging from 0.04 to 0.15 mgRUT/g.	Submerged fermented AYB (FAYB) flour TFC (2.87 mg QE/g) was significantly higher than the native AYB control (2.00 mg QE/g). Raw AYB seed coat exhibited TFC ranging from 0.06 to 0.43 mgRUT/g.	[35]

All values represent approximate % reductions from raw baseline (dry-weight basis) as reported in the cited studies. Most protocols involve multi-step processes that include pre-treatments prior to or during fermentation. These pre-treatments independently contribute significantly to ANF degradation (e.g., leaching during soaking, endogenous enzyme activation during germination, thermal inactivation during boiling) and often account for a substantial portion of the observed reductions. The values therefore reflect combined effects rather than fermentation in isolation.

**Table 3:** Comparison of impact of different fermentation techniques on nutrient bioavailability in Bambara groundnut (BGN) and African yam bean (AYB). A detailed summary of the impacts of spontaneous, controlled, solid-state, and submerged fermentation is provided.

Technique	Microbial Strains	% Antinutrients Reduction	Nutrient Bioavailability	Advantages	Limitations	References
**Spontaneous Fermentation**	Predominantly *Bacillus* species (e.g., *B. subtilis*, *B. cereus*, *B. licheniformis*, *B. pumilus*). Also *Lactobacillus* spp., moulds (*Aspergillus*, *Rhizopus*), and yeasts (*Saccharomyces cerevisiae*) in BGN products.	BGN: Tannin reduced by 34.2–76.4%. Phytic acid was reduced by 18.1–25.6%. Overall, ANF reduction is due to enzyme activation and pH change. AYB: General decrease in phytate.	BGN: Protein increase (3.3–7.4%). Significant increase in minerals (Ba, Ca, Cu, Fe, K, Mg, Mn, P, Zn) due to microbial activity/ANF reduction. IVPD increases substantially (e.g., 70.74% raw to 89.70% fermented BGN).	Traditional, affordable, and common in African condiment production (e.g., dawadawa/iru). Enhances desirable aroma/flavour characteristics.	Unpredictable product quality and nutritional profile. Risk of contamination by spoilage or toxigenic microorganisms (e.g., some *Bacillus cereus* strains). Requires longer fermentation if hulls are present (BGN).	[9]
**Controlled Fermentation (starter-culture-induced)**	Defined starter cultures, often molecularly typed *Bacillus* strains (e.g., *B. subtilis* subsp. *subtilis SFBA3*, *B. amyloliquefaciens* subsp. *plantarum SFBA2*, *B. cereus PALB7*, *B. licheniformis OALB2*) for alkaline condiments. Also, *Lactobacillus* spp. and *Streptococcus* spp. for milk/flours.	BGN: Trypsin inhibitor reduced by 38.3–40.9%. Phytate was reduced by up to 95.9% (depending on strain/method). AYB: Phytic acid reduction of 39.85% (using *S. cerevisiae* SSF). Tannin reduction of 21.43% (using *S. cerevisiae* SSF).	BGN: Improved protein content/quality. Enhanced bioavailability of minerals (K, Mg, P, Zn, Fe). IVPD substantially increased (e.g., to 89.70% in BGN). AYB: Protein content increased (e.g., by 17.3% using *S. cerevisiae* SSF). Amino acids and IVPD increased (e.g., IVPD to 78.18%). Minerals (K, P) significantly increased.	Greater control over process parameters, resulting in enhanced quality and reproducible sensory/nutritional attributes. Potential to ensure microbiological safety by excluding spoilage organisms and eliminating toxins. Can reduce fermentation time (e.g., *B. cereus PALB7* fermentation of BGN at 48 h).	Requires initial characterisation and propagation of starter cultures. May require careful safety assessment for commercial strains (e.g., testing non-toxigenicity of *B. cereus*).	[8,20,33],
**Solid-State Fermentation (SSF: low free water on moist solids)**	Fungal strains (*Rhizopus oligosporus*, *R. nigricans*). Yeast (*Saccharomyces cerevisiae*). Natural flora (for instantaneous germination + SSF of AYB).	BGN: TIA decreased. Oxalate decreased. Phytic acid decreased. AYB: Alkaloid highly reduced (GSFAYB: 0.70% vs. 2.90% native). Tannin was reduced significantly (GSFAYB: 0.48 TAE/100 g).	BGN: Protein content increased (e.g., 18.66% to 22.60% using *Rhizopus*). IVPD enhanced (up to 66.14% increase reported in fungal fermentation). Vitamins (A, riboflavin, niacin) and minerals improved. AYB: Crude fibre increased (GSFAYB: 6.44% vs. 5.01% native). TPC increased (GSFAYB: 2.06 mg GAE/g).	Maximises microbial action by using flour/milled substrate, increasing surface area. An effective method for generating biomass and synthesising enzymes. Enhances crude fibre and phenolic content in AYB/BGN.	Requires careful control of moisture and temperature. May still require prior milling/germination steps (e.g., for optimal AYB SSF).	[3,45]
**Submerged Fermentation (SmF: excess free liquid)**	LAB species (*L. bulgaricus*, *S. thermophilus*, *L. plantarum*) for milk/yoghurt. Natural flora (SmF of whole AYB seeds/FAYB).	AYB: Reduced tannin and alkaloid content in FAYB.	BGN: Improved protein content (e.g., 1.8–2.6% increase in yoghurt). BGN milk supports probiotic growth. AYB: Crude protein increased (FAYB: 23.30% vs. 22.35% native). K and P minerals significantly increased.	Production of highly acceptable liquid products (e.g., BGN milk/yoghurt). Enhanced protein and mineral values in AYB.	Typically requires processing into milk or slurry first (not suitable for solid condiments like dawadawa). Risk of mineral leaching during soaking/processing before SmF.	[3,11]

Definitions follow standard food biotechnology nomenclature. Traditional condiments are classified as SSF due to moist solid substrate and low free water. Yoghurt-like beverages are classified as SmF due to high free liquid. Controlled fermentation (CF) refers to the use of defined starter cultures and can apply to either SSF or SmF. Product examples are mapped based on the dominant process reported in the literature.

## Data Availability

No new data were created or analyzed in this study.
